# Heat shock factor 5 is essential for spermatogenesis in mice: Detected by a new monoclonal antibody

**DOI:** 10.22038/IJBMS.2019.38615.9155

**Published:** 2020-03

**Authors:** Atefeh Hemati, Mohammad Hossein Modarressi, Sedighe Kolivand, Mahnaz Azarnia

**Affiliations:** 1 Department of Cell and Molecular Biology, School of Biological Sciences, Kharazmi University, Tehran, Iran; 2 Department of Medical Genetics, School of Medicine, Tehran University of Medical Sciences, Tehran, Iran; 3 Department of Medical Biotechnology, School of Advanced Technologies in Medicine, Tehran University of Medical Sciences, Tehran, Iran

**Keywords:** Antibody, Development, Hsf5, Hybridoma, Spermatogenesis

## Abstract

**Objective(s):**

Here, we examined the function of our produced monoclonal antibody (mAb10C3) to recognize one of the most important members of the HEAT shock factor family, Hsf5, in embryonic development and in spermatogenic cells of adult mouse testis.

**Materials and Methods:**

The targeting effects of mAb10C3 were investigated by immunohistochemistry analysis in the different phases of the embryo and in the adult testis tissue sections.

**Results:**

The results of immunohistochemistry staining on the mouse embryos by the supernatant of hybridoma clone that produced mAb10C3, in the early and late phases (E7.5 and E14.5) of embryonic development, indicated that mAb10C3 could only detect Hsf5 in E7.5 and it did not have any targeting activity in the late phase of development. Therefore, we showed that the hsf5 gene has expressed in early mouse embryonic development. On the other hand, mAb10C3 could detect Hsf5 in spermatogonia and spermatocytes of adult testis in comparison with a known anti-Hsf5 antibody (ab98939) and an anti-PCNA antibody as a marker of spermatogonia cells.

**Conclusion:**

Taken together, these data indicated that generated anti-testis mAb10C3 was generated against anti-testis proteins, specifically to target Hsf5, and can be useful as a scientific tool to investigate the critical genes in the development and spermatogenesis.

## Introduction

Heat shock factors (Hsfs), as transcription factors, play important roles in development and gametogenesis. Hsf family (Hsf 1-5) has been characterized from vertebrates that regulate responses to environmental stimuli (1-3). The fifth member of the Heat shock factor family (Hsf5) is important in fertility, especially in males (4). Hsf5 is essential for progression of meiotic prophase 1 during spermatogenesis so that *hsf5*^-/-^ mutants are infertile because of gonadal misregulation of several genes (5). 

On the other hand, monoclonal antibody preparation is a useful method for detecting the proteins specifically. Prior to this study, Hsf5, including Hsf1, Hsf2, Hsf4, and Hsf5 have been isolated and characterized from zebrafish. Hsf5 is one of the most important genes that have been selected as suggested by several molecular techniques, though detailed functional characterization of Hsf5 has not been performed in mice (6). Therefore, preparation of anti-Hsf5 monoclonal antibody can be effective in the investigation, prevention, and treatment of experimental male infertility. 

Here, we decided to prepare a specific monoclonal antibody-based hybridoma technology for detection and characterization of Hsf5 in mice. In our laboratory, we have produced and characterized an anti-testis monoclonal antibody. By bioinformatics analysis, we could select several testis-specific proteins that are possibly targets of the desired monoclonal antibody (mAb10C3). 

## Materials and Methods

***Experimental animals***

Balb/C mice were obtained from Tehran University of Medical Sciences, Iran, and maintained under a controlled light cycle (14 L: 10 D). Testes were removed from mice at the ages of 7 and 21 days and 6-8 weeks, rapidly frozen on dry ice and then kept at -80 ^°^C until use. Notably, the treatment of animals was conducted in accordance with the Guiding Principles for the Care and Use of Research Animals promulgated by the Society for the Study of Reproduction (7).

***Generation and screening of the anti-testis monoclonal antibody***

Antibody preparation was performed by cell fusion and hybridoma technology. Hybridoma clones were produced by fusion of SP2/0 cell line and immunized spleen cells of mice. The immunization process of mice was performed five times with 2-week intervals by intraperitoneal injection of the desired antigen (lysates of mice testes) that mixed with Freund’s adjuvant (8, 9). Screening of antibody titer produced by hybridoma clones was performed by indirect ELISA and finally the clone with the highest OD value was selected as the stable hybridoma, called 10C3 clone. The specificity of the antibody produced by hybridoma clone (mAb10C3) was determined by several histological and molecular techniques. The characterization of mAb10C3 was performed on the sperm and testis of mice by immunocytochemistry and immunohistochemistry techniques. In addition, Western blot analysis were performed to determine the size of probable protein(s) that could be target of the produced mAb10C3 (all of the protocols have been explained in our previously published text) (10).

***Bioinformatics analysis***

According to the results of Western blot analysis, mAb10C3 could specifically recognize the mouse testes and sperm proteins that have molecular weights about 53 and 73 KDa, in comparison with other tissue lysates that did not show any band in Western blot. Therefore, our results have suggested that mAb10C3 were specifically prepared against mouse testis antigens. By these data in our previous research, we investigated the most probable target genes of generated mAb on UniGene part of NCBI. As we mentioned previously, Hsf5 was one of the genes that could be the target of desired mAb. Accordingly, we proposed that mAb10C3 was specifically designated against Hsf5 and decided to investigate this proposal here.

***Data analysis of monoclonal antibody 10C3 as the hsf5 marker***

Hsf5 is one of the most important proteins that is specific target of mAb10C3. So here, we investigated how mAb10C3 could act as anti-Hsf5 antibody on the testis tissue of mice. In this regard, the specificity of the antibody to recognize the Hsf5 protein was demonstrated through immunohistochemistry on the embryo and adult testis of mice. 

***Immunostaining analysis***

For immunohistochemically (IHC) analyses, testes from adult Balb/C mice were isolated and fixed in PBS containing 4% paraformaldehyde in pH 7.4 for 18–24 hr at room temperature (depending on the size of tissue) and embedded in paraffin wax. The samples were cut into 2–5 µm sections using a cryotome (Leica Bio systems) and collected on glass slides. Then dewaxing in a series of ethanol, antigen retrieval was performed by Tris/EDTA (pH=9) and Heat-induced (HIER). After blocking with 1% BSA in TBS for 2 hr at room temperature, the slides were incubated with mAb10C3 and anti-Hsf5 antibody diluted in PBS containing 1% BSA and 0.1% Triton X at 1:1000 (anti-Hsf5) and 1:100 (mAb10C3) dilutions, overnight at 4 °C. FITC-conjugated goat polyclonal anti-mouse IgG (Abcam, USA) at 1:2,000 dilution was used as a secondary antibody to incubate for 2 hr at room temperature followed by DAPI (Calbiochem) nuclear staining for 5 min. The slides were mounted in Murray’s clear solution and the image was captured using an Optika XDS-2 inverted microscope (11). 

Then, IHC protocol was performed on the mouse embryo sections in the early and late phases of development. Anti-PCNA (a proliferation marker) antibody staining, as the control of prepared mAb10C3, was used to identify different phases of cellular population during embryonic development of mice. 

Embryos were removed at E7.5 and E14.5 embryonic ages from pregnant mice and washed three times, 5 min each, in a 50-ml drop of PBS supplemented with polyvinylpyrrolidone (PBS-PVP, 4 mg/ml). This was followed by fixation at room temperature for 5 min in neutral buffered formalin (3.7%), washing, and fixation for 10 min in 70% ethanol. For membrane permeabilization, embryos were incubated in 0.1% Triton X-100 in PBS for 5 min. Nonspecific binding of antibodies was suppressed by incubation in a blocking solution (0.1% BSA in PBS). Thereafter, embryos were transferred for 1 hr into the produced monoclonal antibody (mAb10C3) and also anti-PCNA antibody (Dako, Denmark) diluted 1:100 in blocking solution. Specimens were washed three times for 5 min each time in PBS-PVP. For visualization, embryos were incubated for 1-2 hr in the presence of fluorescein isothiocyanate (FITC)-labeled anti-mouse immunoglobulins (Dako A/S) at a dilution of 1:50 in blocking solution. No reaction was observed when the primary antibody was omitted. The washed embryos were placed onto a coverslip and immediately mounted in 10 ml of 1% agar dissolved in a solution containing 100 mg 1, 4-diazabicyclo (2.2.2) octane and 50% glycerol in PBS. Before use, the agar was melted in a microwave oven. The coverslip was set on an object glass to rest on supports made by drops of nail polish. Finally, glycerol diluted with PBS (2:1) was applied under the coverslip to fill the remaining spaces between the coverslip and the object-glass. The attachment of the coverslip was secured with nail polish. Specimens were stored at -22 ^°^C until evaluation.

## Results

***Identification of Hsf5 protein in adult testis by generated mAb10C3***

**Figure 1:**
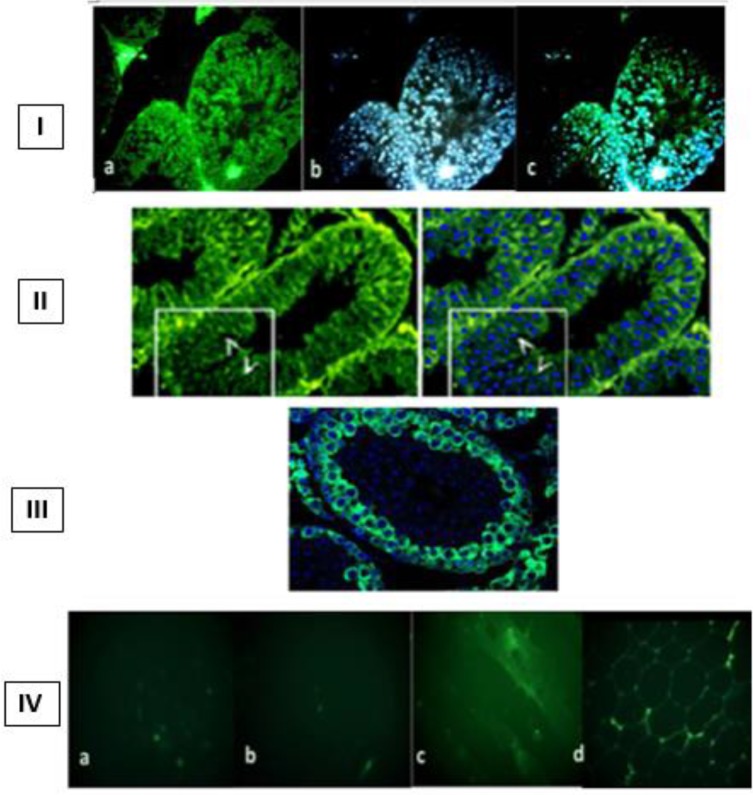
Immuno-localization of heat shock factor 5 (Hsf5) on adult testis. Sections from adult mouse testes were stained with the generated mAb10C3 (I), anti-Hsf5 marker (II), and anti-PCNA (III) antibodies. The data were compared using immunohistochemistry staining on several tissue sections such as liver (a), kidney (b), muscle (c), and skin (d) (IV) to show the specificity of mAb10C3 (Scale bar = 200 µm)

In our previous research, the results of IHC techniques indicated that the localization of mAb10C3 was only on the spermatogonia and spermatocyte membranes specifically; whereas other tissue sections from the liver, kidneys, skin, and muscles, as the negative control groups, were not significantly detected by desired mAb10C3. Here, we demonstrated that mAb10C3 could identify specifically Hsf5, in adult testis tissue sections of mice by immunohistochemistry staining in comparison with a known anti-Hsf5 marker and the anti-PCNA antibody. The results indicated that mAb10C3 can detect the Hsf5 protein on testis sections of mice and this activity is similar to other anti-Hsf5 antibodies that were produced before. In addition, the localization of mAb10C3 was specifically on the spermatogonia cells in comparison with the control group (usage of anti-PCNA primary antibody). The immunostaining results were shown in Figure 1. In this regard, we suggest that the desired mAb10C3 was designated against Hsf5 protein of testis tissue sections. 

***Identification of Hsf5 protein in mouse embryonic development by generated mAb10C3***

**Figure 2.**
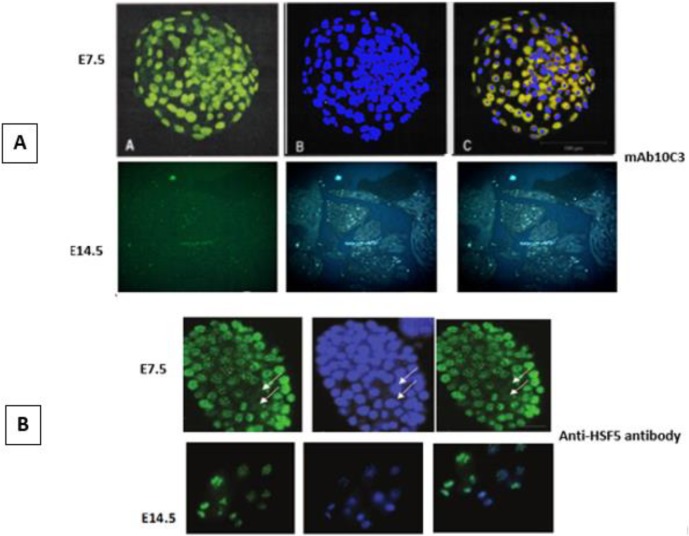
Immuno-localization of heat shock factor 5 (Hsf5) on the mouse embryonic development by the generated monoclonal antibody (mAb10C3) (A), in comparison with a known anti-Hsf5 antibody (ab98939), as a positive control, (B). As shown in the figure, mAb10C3 can detect Hsf5 at mouse embryo E7.5 and no targeting effect was shown at E14.5, similar effect as in control group that used another anti-Hsf5 antibody. Scale bar = 100 µm

In order to detect the activity of produced mAb10C3 on different phases of embryonic development and especially to detect the Hsf5 protein in mouse embryo, we performed the immuno-localization of Hsf5 by desired mAb10C3 on the early and late phases of development. Analysis of molecular function and localization of protein products of Hsf5 that were targeted by our designated antibody, revealed that mAb10C3 could specifically detect Hsf5 protein on the early phases of mouse embryonic development, at E7.5, not at the late phases of development, E14.5 (Figure 2A), these results were compared with a known anti-Hsf5 antibody (ab98939) (Figure 2B).

## Discussion

The complex series of cellular signaling molecules and transcriptional factors are involved in differentiation and development of mammalian male sexual gonads, testes; and the regulated and complicated genetic network is the basis of this molecular pathway, generally (12). During mouse embryogenesis, the bipotential gonad appears at embryonic day E10.5, firstly. Several critical genes are involved in this period of gonadogenesis (13, 14). In summary, identification of testis development genes (TDG) in mammalians could be helpful in functional characterization of each stage of the development of this sexual gland and could eliminate the occurrence of defects in each phase of testis development (15).

Spermatogenesis is a process during which spermatogonia cells (diploid germ cells) are converted into spermatozoa (haploid cells) and the cell types of this diploid to haploid transition include spermatogonia (2n), spermatocytes (2n), spermatids (n), and spermatozoa (n) (16). The complex series of progressive changes and signaling pathways are involved in spermatogenesis in mammalians. In mammals, testicular temperature is lower than normal body temperature, because spermatogenesis needs a stable temperature. The vulnerable nature of mammalian spermatogenesis to thermal insult has been known by many scientists. The primary spermatocytes were shown to be most sensitive to increases in temperature; thus, an addition in testicular temperature has a destructive effect on spermatogenesis and can increase the risk of infertility in males (17). Several possible mechanisms of germ cell death by thermal injury include decreases in the synthesis of DNA, RNA and proteins in germ cells, decreased capillary blood flow that supplies germ cells, and indirect effects of proteins synthesized by Sertoli and Leydig cells (18). However, the molecular events underlying the activation of germ cell death remain poorly understood.

The most heat-sensitive spermatocytes are primary spermatocytes during the first meiotic, as there are many factors to advance first meiotic division of spermatogenesis. The large family of proteins that has received considerable attention is the heat shock protein (hsp) family (19). 

In addition to promoting cell survival under stressful conditions, heat shock factors are involved in the progression of cancer. The Hsf family is also important for developmental processes such as gametogenesis, neurogenesis, and maintenance of sensory organs (20, 21). 

In mammals, the Hsf gene family consists of three members, which are highly conserved. All of the Hsf family members are expressed in testes of mammalians, usually in spermatocytes and round spermatids (22), suggesting the involvement of the Hsf system in normal spermatogenesis.

Hsf5 is one of the specific members of this family that is expressed exclusively in the testes and has a critical role in spermatogenesis while expression of the other members is more ubiquitous (23, 24).

Accordingly, we decided to recognize Hsf5 as a testis-specific gene. Here, we described an uncharacterized anti-heat shock factor 5 (Hsf5 from mice) monoclonal antibody which is required to investigate the spermatogenesis process. Accordingly, in our laboratory, the monoclonal antibody was produced against testis antigens of mice by “hybridoma technology”. The prepared antibody showed high specificity for the proteins that are encoded from critical genes involved in the early development of testis in both embryo and adult mouse testis tissue.

We describe the functional characterization of our prepared monoclonal antibody (mAb10C3) to target Hsf5, with bona fide function in germ cell development and the critical role of Hsf5 in meiotic progression of spermatogenesis in mice. 

In order to confirm our proposal, the targeting effects of prepared monoclonal antibody (mAb10C3) were investigated on the mouse embryo and adult testis by the immunohistochemistry technique in comparison with the positive antibody controls (25). Comparing with our gold standards (both sensitivity and specificity of the designed antibody), mAb10C3 can be used as a specific monoclonal antibody to detect the critical antigens that are involved in male germ cell development.

Future analysis of the effect of this antibody to detect the essential antigens that are possibly involved in germ cell development might provide more information about additional functions of the generated monoclonal antibody.

## Conclusion

Our data showed that the produced monoclonal antibody is a reliable antibody for testis antigen detection. Therefore, we recommend this antibody to be used as a marker of spermatogenesis in the biological and medical studies. 
